# Supplementary material for: Characterization of Peripheral Blood TCR in Patients with Type 1 Diabetes Mellitus by BD RhapsodyTM VDJ CDR3 Assay
Source: Cells. 2022 May 12;11(10):1623. doi: 10.3390/cells11101623 (PMC9139223; doi:10.3390/cells11101623)
Supplement: Supplementary file 1 [file cells-11-01623-s001.zip › cells-1686212-supplementary.pdf]

Supplementary Table S1. Clinical characteristics of the study patients

Sample	1	2	3	4
Sex	Male	Female	Male	Male
Type	1A	1B	1B	SPIDDM
Age, yrs	71	68	47	49
Disease duration	16	46	19	7
Height, cm	169	150.2	181	168.4
Body weight, kg	57	45	100	76
Body mass index, kg/m^2	20.0	19.9	30.5	26.8
Fasting plasma glucose, mmol/L	7.2	4.3	11.3	7.3
Hemoglobin A1c, %	8.1	7.5	7.5	8.5
C-peptide, mmol/L	< 0.01	< 0.01	< 0.01	0.301
Creatinine, mmol/L	65.4	66.3	86.6	84.9
Estimated GFR, mL/min/1.73m^2	79.4	58.5	66.1	66.8
Urine albumin to creatinine ratio, mg/gCr	3673	64	8	19
Anti-GAD antibody	237	<5	<5	18.1

Cr, creatinine; GAD, Glutamic Acid Decarboxylase; GFR, glomerular filtration rate; SPIDDM, slowly progressive insulin-dependent diabetes mellitus.

Supplementary Table S2. Shannon-index H'

	TRA	TRB
S1	10.80	10.83
S2	11.62	11.69
S3	10.26	10.57
S4	11.37	11.25
S1 FOXP3+	5.37	5.57
S2 FOXP3+	6.27	6.35
S3 FOXP3+	5.62	6.14
S4 FOXP3+	5.84	5.77
S1 CD8+	9.17	9.14
S2 CD8+	9.74	9.77
S3 CD8+	8.24	8.61
S4 CD8+	9.44	9.37

TRA, T cell receptor alpha; TRAB, T cell receptor beta.

Supplementary Table S3. TCR clonotype of CD8 positive cells

	Clone ID	Frequency (%)	TRA			TRB		
			TRAV	CDR3	TRAJ	TRBV	CDR3	TRBJ
S1	1-1	7.9	V27	AGAISNNDMR	J43	V9	ASSVVGSGTDEQF	J2-1
	1-2	7.4	V13-1	AASGSSASKII	J3	V6-5	ASSYSGQGSYT	J1-2
	1-3	4.0	V17	ATDSGGYQKVT	J13	V19	ASRLTGAGANVLT	J2-6
	1-4	3.6	V1-1	AVRDLDGGFKTI	J9	V10-3	AISEPEGNTEAF	J1-1
	1-5	3.4	V14/DV4	AMRRPSGGYNKLI	J4	V19	ASNAGYNEQF	J2-1
	1-6	3.1	V13-1	AASWDNAGNMLT	J39	V12-3	ASSDGTGGYEQY	J2-7
	1-7	2.7	V12-2	AVNPRRGFKTI	J9	V27	ASSLGLAGGYEQF	J2-1
	1-8	2.6	V6	ARASYGGATNKLI	J32	V9	ASSVTFERVPGANVLT	J2-6
	1-9	2.6	V1-1	APDTGRRALT	J5	V20-1	SARVVTGSSYEQY	J2-7
	1-10	2.6	V17	ATDMEEGGSQGNLI	J42	V19	ASNAGYNEQF	J2-1
S2	2-1	18.1	V12-1	VVRARPPLPWSGGGADGLT	J45	V7-2	ASTPPSSPGYEQY	J2-7
	2-2	10.3	V12-3	VPGGSASKII	J3	V20-1	SARGRPAGEQF	J2-1
	2-3	9.1	V6	ALKGYSGGYQKVT	J13	V28	ASSFSDRVNQPQH	J1-5
	2-4	8.1	V17	ATEGDSNYQLI	J33	V7-3	ASSSGTGDSLH	J1-6
	2-5	5.6	V12-3	AMSDYGGATNKLI	J32	V5-1	ASSPGRDRGSYEQY	J2-7
	2-6	5.2	V21	AVSPLSSGSARQLT	J22	V7-2	ASSLVSGPTYEQY	J2-7
	2-7	4.8	V9-2	AFDGGGATNKLI	J32	V4-2	ASSPGLGQPQH	J1-5
	2-8	4.4	V5	AESSGTGKLI	J37	V24-1	ATSDPAGGRADTQY	J2-3
	2-9	4.3	V12-1	VVNPRGSTLGRLY	J18	V10-2	ASSAGQGEAF	J1-1
	2-10	3.5	V14/DV4	AMQIDSWGKLQ	J24	V29-1	SVEDPHMDTQY	J2-3
S3	3-1	5.6	V38-1	AFSGGYQKVT	J13	V7-9	ASSLAGEGSGTGELF	J2-2
	3-2	3.4	V2	AVEDLLNSGYSTLT	J11	V6-2	ASSLRDSSYEQY	J2-7

	3-3	3.3	V21	AQGAYKLS	J20	V7-6	ASSPREAYEQY	J2-7
	3-4	2.7	V14/DV4	AMREGGSGYSTLT	J11	V2	ASSDRRGSSTDTQY	J2-3
	3-5	2.5	V27	GLN	J41	V20-1	SALRSGELF	J2-2
	3-6	2.4	V12-3	AMSGNQFY	J49	V28	ASRRFTGTDTQY	J2-3
	3-7	2.3	V12-3	AMTAGTYKYI	J40	V29-1	SADSSVGFHNEQF	J2-1
	3-8	2.3	V14/DV4	AMREYGNQFY	J49	V5-4	ASSRGQQPSYEQY	J2-7
	3-9	2.2	V12-2	AVNNQAGTALI	J15	V4-3	ASSQDLGANTEAF	J1-1
	3-10	2.1	V38-2/DV8	AYRSRGDMR	J43	V27	ASSFLAGATGELF	J2-2
S4	4-1	9.3	V10	VVSAFFSGGSYIPT	J6	V5-1	ASSSSRDRGNYEQY	J2-7
	4-2	5.5	V21	AVKGGSEKLV	J57	V7-8	ASSLVGLESYNEQF	J2-1
	4-3	3.7	V12-1	AVNLNTGFQKLV	J8	V2	ASRGYSYEQY	J2-7
	4-4	3.1	V12-3	AMVRAGGYNKLI	J4	V6-6	ASRSERESPISNEQF	J2-1
	4-5	3.1	V5	AALSGGSYIPT	J6	V4-3	ASSQGLREGLGEQY	J2-7
	4-6	3.1	V14/DV4	AMRNKSWGKFQ	J24	V3-1	ASSQEIVRTSGENTGELF	J2-2
	4-7	3.0	V6	ALGHSSASKII	J3	V20-1	SARDRDSSSYEQY	J2-7
	4-8	2.9	V21	AVASNFGNEKLT	J48	V29-1	SVAAGAQTQY	J2-5
	4-9	2.3	V2	AVEERIMGTYKYI	J40	V20-1	SARGVAANPYEQY	J2-7
	4-10	2.3	V12-1	VVPYNTDKLI	J34	V5-6	ASKPPGGSIYEQY	J2-7

CDR3, complementarity-determining region 3; TRA, T cell receptor alpha; TRAJ, TRA joining; TRAV, TRA variable; TRB, T cell receptor beta; TRBJ, TRB variable; TRBV, TRB variable.

Supplementary Table S4. TCR clonotype of FOXP3 positive cells

	Clone ID	Frequency (%)	TRA			TRB		
			TRAV	CDR3	TRAJ	TRBV	CDR3	TRBJ
S1	1-1	6.7	V12-3	AMRFKSGYNKLI	J4	V18	ASSPPTSGASYEQY	J2-7
	1-2	6.3	V12-2	AVNIRDSSYKLI	J12	V20-1	SARSRLAVSGELF	J2-2
	1-3	6.0	V12-3	AMSDSGGGADGLT	J45	V3-1	ASSQRGGTQY	J2-3
	1-4	5.9	V12-1	VGLTNAGKST	J27	V11-2	ASSLGTQTTNEKLF	J1-4
	1-5	5.6	V2	AVEGGSGNTGKLI	J37	V2	ASSEEGNTEAF	J1-1
	1-6	4.6	V9-2	ATTRYSGAGSYQLT	J28	V28	ASTGTTSINEQY	J2-7
	1-7	4.1	V16	A*RNFGNEKLT	J48	V12-3	ASSSRGGDNQPQH	J1-5
	1-8	3.2	V25	GRSGSARQLT	J22	V30	AWNRQGANTGELF	J2-2
	1-9	3.1	V13-1	AAPTIGRSKLT	J56	V7-3	ASSPLSSGANVLT	J2-6
	1-10	3.0	V4	LVAFDTGRRALT	J5	V23-1	ASSPPKFELLRAV	J2-7
S2	2-1	17.2	V9-2	ALSSNDYKLS	J20	V12-3	ASTLDGPGSPLH	J1-6
	2-2	9.3	V9-2	ALSGRNTGGFKTI	J9	V2	ASSRTKTDTQY	J2-3
	2-3	7.2	V35	AGPYSGAGSYQLT	J28	V28	ASSPSSGRASYEQY	J2-7
	2-4	5.4	V41	AVNAGNMLT	J39	V7-9	ASSSLDRGNIQY	J2-4
	2-5	4.5	V13-1	AASRPQGRRC*RTH	J45	V7-9	ASRLDATNEKLF	J1-4
	2-6	4.3	V38-2/DV8	AYRSYGAGNMLT	J39	V28	ASSQQGRQETQY	J2-5
	2-7	3.5	V12-1	VVRLNTGGFKTI	J9	V20-1	SARVGSTEKLF	J1-4
	2-8	2.9	V41	AVSSTPARQLT	J22	V6-6	ASSYSGSGSRRWHEQY	J2-7
	2-9	2.8	V38-2/DV8	APLGAGSYQLT	J28	V20-1	SASLMAVSYEQY	J2-7
	2-10	2.5	V12-1	VVNKQTGANNLF	J36	V28	ASRRRGGGTGELF	J2-2
S3	3-1	3.2	V21	GFSSGSARQLT	J22	V7-2	ASSFGRYEQY	J2-7
	3-2	2.6	V22	AANTPLV	J29	V12-3	ASSLLVDTQY	J2-3

	3-3	2.5	V21	AVTTGKST	J27	V20-1	SGQGTDTQY	J2-3
	3-4	2.1	V12-1	VVNMGGGFKTI	J9	V20-1	SASGGPGYNEQF	J2-1
	3-5	2.1	V13-1	AAGPMDSSYKLI	J12	V6-1	ASRLALTYNEQF	J2-1
	3-6	1.9	V13-1	AARGTSYGKLT	J52	V20-1	SARDPSSGLYNEQF	J2-1
	3-7	1.7	V21	AVRDDYKLS	J20	V20-1	SAGPGLAGVYEQF	J2-1
	3-8	1.6	V6	ALEDTGRRALT	J5	V25-1	ASTAPLGGLKQY	J2-3
	3-9	1.5	V21	AVYTSGSARQLT	J22	V6-5	ASSQGGGNTIY	J1-3
	3-10	1.5	V9-2	ALISSGSARQLT	J22	V10-2	ASSESRGSSNQPQH	J1-5
S4	4-1	4.4	V13-1	AAGRGNNRLA	J7	V12-3	ASSRTGGGYGYT	J1-2
	4-2	4.3	V10	VVRIAAISNTGKLI	J37	V24-1	ATSDHTQGRQGYT	J1-2
	4-3	3.8	V12-2	AVNGENFNKFY	J21	V12-3	ASSLAGTGVGYT	J1-2
	4-4	3.8	V2	AVEDRRQSGAGSYQLT	J28	V28	ASSFGFSNTEAF	J1-1
	4-5	3.8	V13-1	AASMNNQGGKLI	J23	V3-1	ASSQVRTGAYSNQPQH	J1-5
	4-6	3.7	V13-1	AASHGGSQGNLI	J42	V9	ASSVEVSGSYNEQF	J2-1
	4-7	3.4	V21	AGYNNDMR	J43	V4-1	ASSQGQGNYGYT	J1-2
	4-8	3.1	V1-1	ADRMDSNYQLI	J33	V20-1	SASPGQGADTQY	J2-3
	4-9	2.9	V12-2	AVRTKGGYQKVT	J13	V20-1	SPRGGGTEAF	J1-1
	4-10	2.8	V13-1	AASHGGSQGNLI	J42	V27	ASSYGVGGSIQY	J2-4

CDR3, complementarity-determining region 3; TRA, T cell receptor alpha; TRAJ, TRA joining; TRAV, TRA variable; TRB, T cell receptor beta; TRBJ, TRB variable; TRBV, TRB variable.

Supplementary Table S5. Top five CDR3 motif of TRA in CD8+ cells and clustering

S1	Total	1	2	3	4	5	6	7	8	9	10
1	31	3	0	0	0	21	5	0	0	1	1
2	75	4	1	0	0	34	34	0	2	0	0
3	12	0	0	0	0	8	3	0	1	0	0
4	13	0	0	0	0	9	3	0	1	0	0
5	85	5	1	0	1	52	26	0	0	0	0

S2	Total	1	2	3	4	5	6	7	8	9	10	11
1	35	0	1	2	0	0	26	1	0	1	1	3
2	180	1	1	4	0	0	64	104	0	0	0	6
3	10	0	0	0	0	0	7	1	1	0	0	1
4	13	0	0	0	0	0	11	2	0	0	0	0
5	399	2	6	22	0	0	296	51	1	1	3	17

S3	Total	1	2	3	4	5	6	7	8	9	10	11	12	13	14	15	16	17	18	19
1	61	0	2	2	22	12	18	1	0	0	1	2	0	1	0	0	0	0	0	0
2	90	1	1	3	25	12	42	1	0	3	0	1	0	0	1	0	0	0	0	0
3	20	0	0	0	1	4	1	1	1	11	0	0	0	0	1	0	0	0	0	0
4	17	0	0	0	7	5	5	0	0	0	0	0	0	0	0	0	0	0	0	0
5	436	6	6	15	65	72	180	20	3	45	2	12	1	7	2	0	0	0	0	0

S4	Total	1	2	3	4	5	6	7	8	9	10	11
1	14	1	2	0	0	1	9	0	0	1	0	0
2	123	4	7	0	5	41	61	2	0	3	0	0
3	16	0	0	0	1	9	4	0	1	0	1	0

4	81	0	0	0	4	36	39	1	0	1	0	0
5	548	5	25	5	8	186	297	5	1	9	3	4

CDR3, complementarity-determining region 3; TRA, T cell receptor alpha; TRB, T cell receptor beta.

Supplementary Table S6. Top five CDR3 motif of TRB in CD8+ cells and clustering

S1	Total	1	2	3	4	5	6	7	8	9	10
1	648	16	43	0	5	194	358	2	0	21	8
2	16	0	0	0	3	11	2	0	0	0	0
3	19	1	1	0	0	5	12	0	0	0	0
4	63	2	1	0	1	18	38	0	0	0	3
5	7	0	0	0	0	0	6	1	0	0	0

S2	Total	1	2	3	4	5	6	7	8	9	10	11
1	883	7	10	50	0	1	596	163	3	0	10	42
2	9	0	2	0	0	0	2	3	0	0	0	2
3	0	0	0	0	0	0	0	0	0	0	0	0
4	125	2	1	4	0	0	108	4	0	0	3	3
5	0	0	0	0	0	0	0	0	0	0	0	0

S3	Total	1	2	3	4	5	6	7	8	9	10	11	12	13	14	15	16	17	18	19
1	499	8	11	5	186	180	2	0	3	104	0	0	0	0	0	0	0	0	0	0
2	1	0	0	1	0	0	0	0	0	0	0	0	0	0	0	0	0	0	0	0
3	14	3	1	0	6	4	0	0	0	0	0	0	0	0	0	0	0	0	0	0
4	55	0	0	2	16	25	0	0	0	12	0	0	0	0	0	0	0	0	0	0
5	21	1	2	1	11	5	0	0	0	1	0	0	0	0	0	0	0	0	0	0

S4	Total	1	2	3	4	5	6	7	8	9	10	11
1	647	16	43	0	5	194	358	2	0	21	8	0
2	16	0	0	0	3	11	2	0	0	0	0	0
3	24	3	2	0	0	5	14	0	0	0	0	0

4	65	3	1	0	1	18	42	0	0	0	3	0
5	35	1	5	0	4	15	10	0	0	0	0	0

CDR3, complementarity-determining region 3; TRA, T cell receptor alpha; TRB, T cell receptor beta.

Supplementary Table S7. Top five CDR3 motif of TRA in FOXP3+ cells and clustering

S1	Total	1	2	3	4	5	6	7	8	9	10
1	2	2	0	0	0	0	0	0	0	0	0
2	2	1	0	0	0	1	0	0	0	0	0
3	2	1	0	0	0	1	0	0	0	0	0
4	24	7	0	0	3	11	3	0	0	0	0
5	4	3	0	0	0	0	1	0	0	0	0

S2	Total	1	2	3	4	5	6	7	8	9	10	11
1	8	0	1	0	2	0	0	0	5	0	0	0
2	5	0	0	0	1	0	0	0	4	0	0	0
3	8	0	2	0	0	0	0	0	6	0	0	0
4	26	1	2	0	1	0	0	0	22	0	0	0
5	2	0	1	0	0	0	0	0	1	0	0	0

S3	Total	1	2	3	4	5	6	7	8	9	10	11	12	13	14	15	16	17	18	19
1	11	1	0	0	0	0	1	0	0	0	0	0	0	0	9	0	0	0	0	0
2	18	0	0	2	0	0	1	0	0	0	0	1	1	0	13	0	0	0	0	0
3	9	0	0	3	0	0	0	0	0	0	0	0	1	0	5	0	0	0	0	0
4	39	0	0	3	0	0	2	0	0	0	0	1	3	2	28	0	0	0	0	0
5	6	0	0	3	0	0	0	0	0	0	0	1	0	0	2	0	0	0	0	0

S4	Total	1	2	3	4	5	6	7	8	9	10	11
1	8	0	7	0	0	0	0	0	0	0	1	0
2	8	0	7	0	0	0	0	0	0	0	1	0
3	3	0	2	0	0	0	0	0	0	0	1	0

4	22	2	17	0	0	0	0	0	0	0	3	0
5	2	0	1	0	0	0	0	0	0	0	1	0

CDR3, complementarity-determining region 3; TRA, T cell receptor alpha; TRB, T cell receptor beta.

Supplementary Table S8. Top five CDR3 motif of TRB in FOXP3+ cells and clustering

S1	Total	1	2	3	4	5	6	7	8	9	10
1	6	0	2	0	0	1	2	0	0	1	0
2	89	3	69	0	0	4	2	0	0	3	8
3	1	0	1	0	0	0	0	0	0	0	0
4	3	0	2	0	0	0	1	0	0	0	0
5	5	0	4	0	0	0	0	0	0	0	1

S2	Total	1	2	3	4	5	6	7	8	9	10	11
1	2	0	1	1	0	0	0	0	0	0	0	0
2	113	4	8	1	3	0	0	0	96	0	0	1
3	2	1	0	0	0	0	0	0	1	0	0	0
4	2	0	1	0	0	0	0	0	1	0	0	0
5	5	1	0	0	0	0	0	0	4	0	0	0

S3	Total	1	2	3	4	5	6	7	8	9	10	11	12	13	14	15	16	17	18	19
1	6	0	0	1	0	0	0	0	0	0	0	0	0	1	4	0	0	0	0	0
2	163	0	0	35	0	0	4	0	0	0	0	7	4	7	106	0	0	0	0	0
3	2	0	0	0	0	0	0	0	0	0	0	1	0	0	1	0	0	0	0	0
4	7	0	0	2	0	0	0	0	0	0	0	0	0	1	4	0	0	0	0	0
5	3	0	0	0	0	0	0	0	0	0	0	0	0	0	3	0	0	0	0	0

S4	Total	1	2	3	4	5	6	7	8	9	10	11
1	6	0	2	0	0	1	2	0	0	1	0	0
2	89	3	69	0	0	4	2	0	0	3	8	0
3	1	0	1	0	0	0	0	0	0	0	0	0

4	3	0	2	0	0	0	1	0	0	0	0	0
5	5	0	4	0	0	0	0	0	0	0	1	0

CDR3, complementarity-determining region 3; TRA, T cell receptor alpha; TRB, T cell receptor beta.

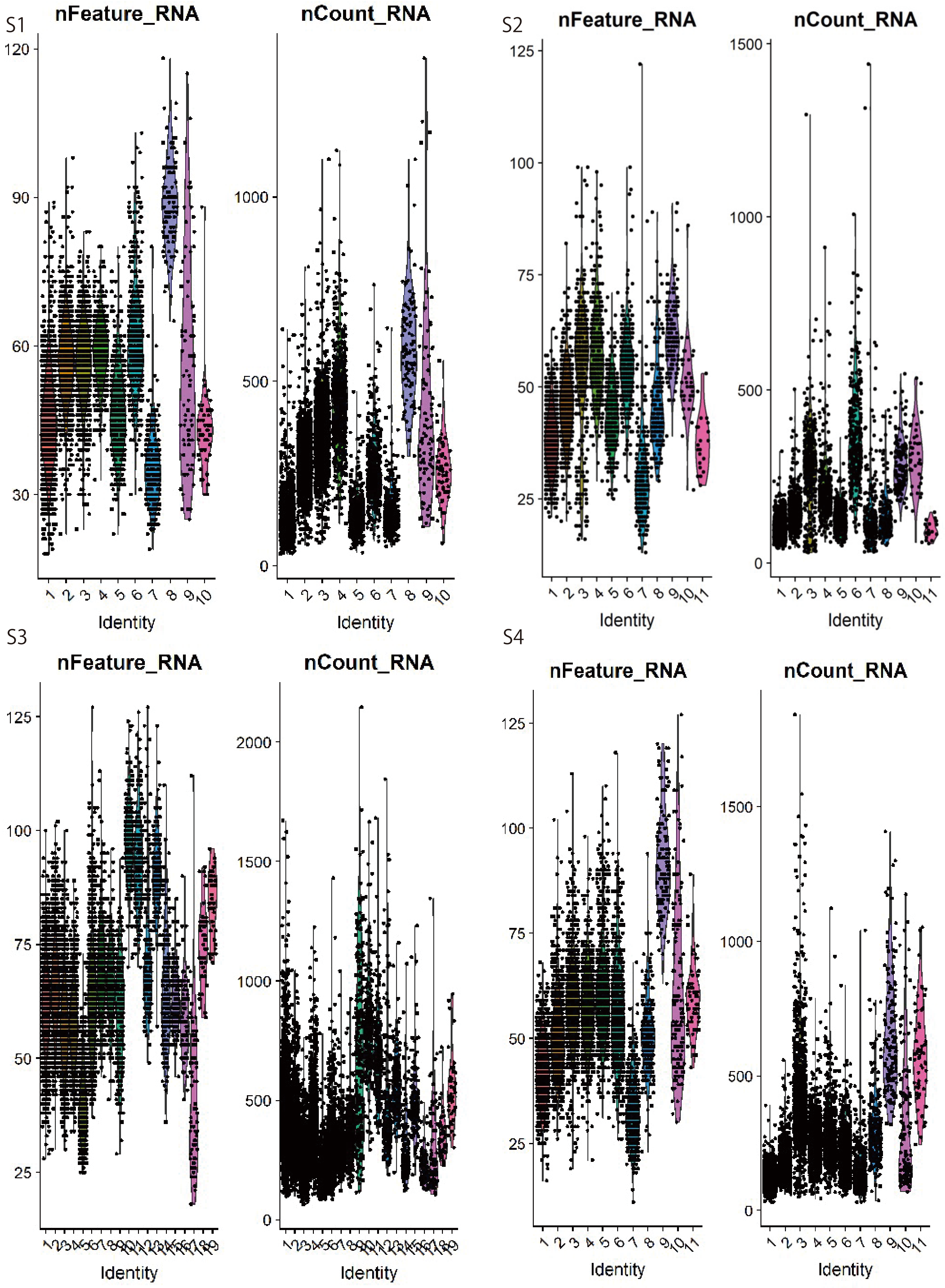

Supplementary Figure S1

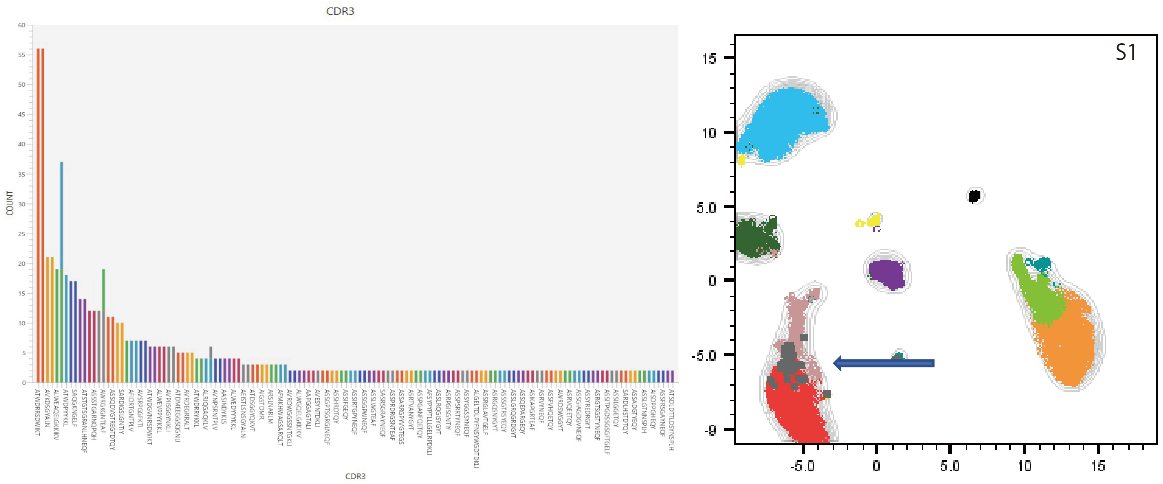

Cluster name	Count
Cluster 10 Unknown	57.0
Cluster 9 Unknown	79.0
Cluster 8 Unknown	98.0
Cluster 7 Unknown	330
Cluster 6 GZMK+ T8m cells	498
Cluster 5 CD8B+ T8n cells	600
Cluster 4 Unknown	631
Cluster 3 Unknown	1597
Cluster 2 KLRF1+ NK cells	1650
Cluster 1 IL7R+ T4 cells	1840
Ungated	7380

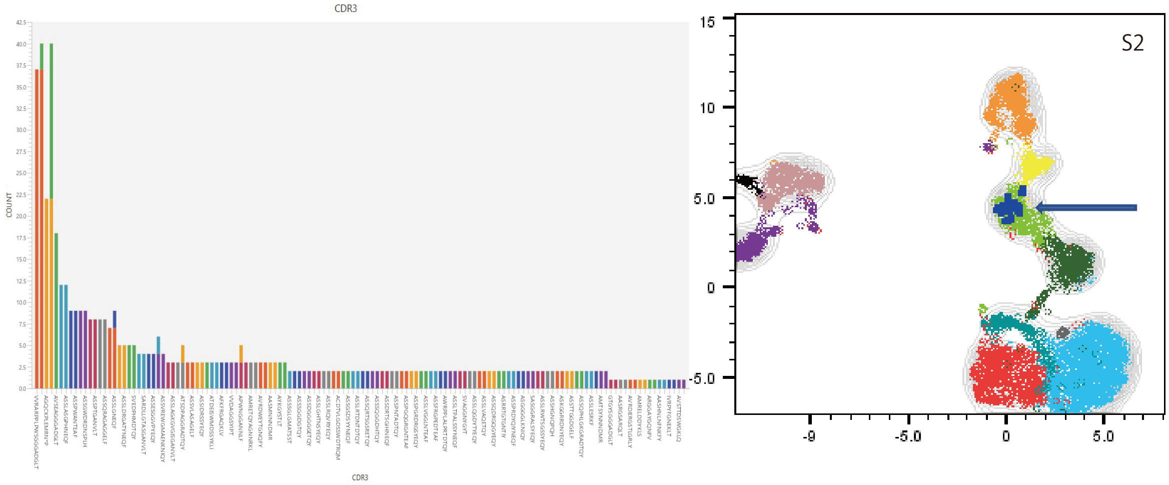

Cluster name	Count
Cluster 11 IL7R+ T4 cells	21.0
Cluster 10 Unknown	39.0
Cluster 9 CD8B+ T8n cells	141
Cluster 8 FOXP3+ Treg cells	177
Cluster 7 Unknown	221
Cluster 6 Unknown	318
Cluster 5 CD8B+ T8n cells	342
Cluster 4 GZMK+ T8m cells	351
Cluster 3 KLRF1+ NK cells	369
Cluster 2 IL7R+ T4 cells	660
Cluster 1 IL7R+ T4 cells	1233
Ungated	3872

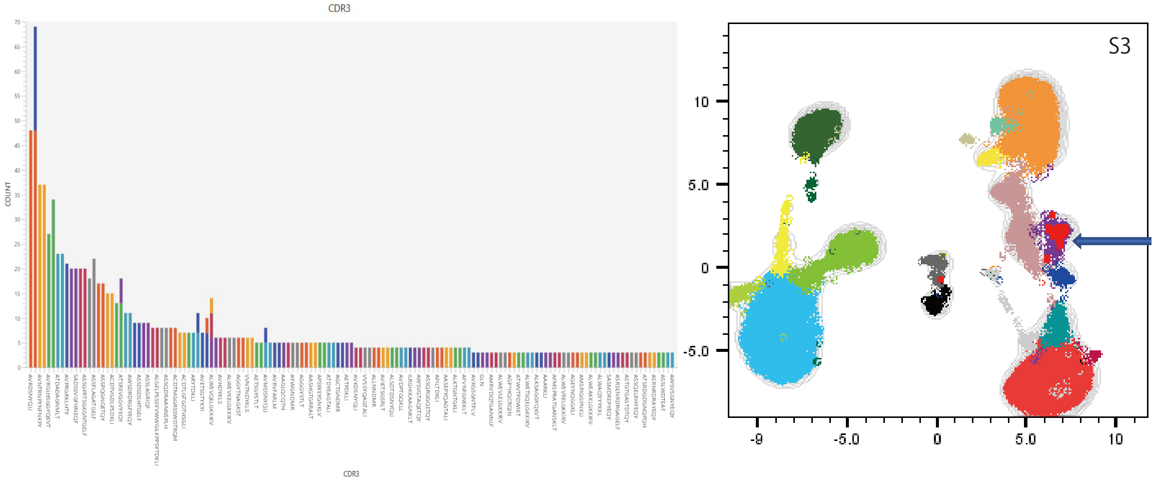

Cluster name	Count
Cluster 19 KLRF1+ NK cells	44.0
Cluster 18 IL7R+ T4 cells	48.0
Cluster 17 Unknown	71.0
Cluster 16 IL7R+ T4 cells	74.0
Cluster 15 Unknown	148
Cluster 14 FOXP3+ Treg cells	162
Cluster 13 Unknown	169
Cluster 12 KLRF1+ NK cells	197
Cluster 11 Unknown	211
Cluster 10 KLRF1+ NK cells	247
Cluster 9 Unknown	275
Cluster 8 KLRF1+ NK cells	400
Cluster 7 GZMK+ T8m cells	404
Cluster 6 Unknown	1125
Cluster 5 Unknown	1141
Cluster 4 Unknown	1168
Cluster 3 IL7R+ T4 cells	3129
Cluster 2 KLRF1+ NK cells	3861
Cluster 1 Unknown	4050
Ungated	16924

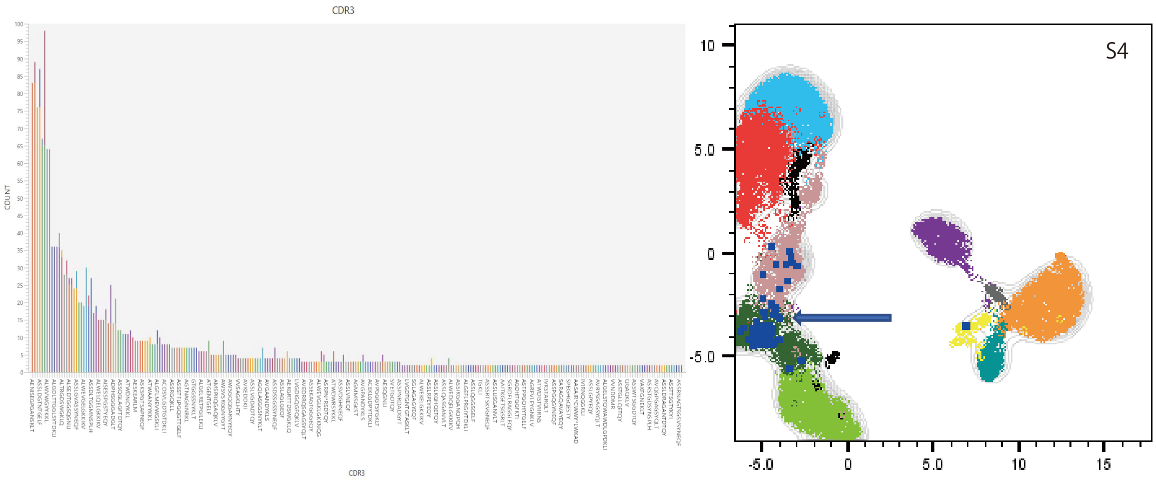

Cluster name	Count
Cluster 11 Unknown	64.0
Cluster 10 Unknown	130
Cluster 9 Unknown	136
Cluster 8 Unknown	245
Cluster 7 Unknown	471
Cluster 6 Unknown	735
Cluster 5 Unknown	803
Cluster 4 KLRF1+ NK cells	1098
Cluster 3 Unknown	1174
Cluster 2 Unknown	1283
Cluster 1 IL7R+ T4 cells	1437
Ungated	7576

Supplementary Figure S2

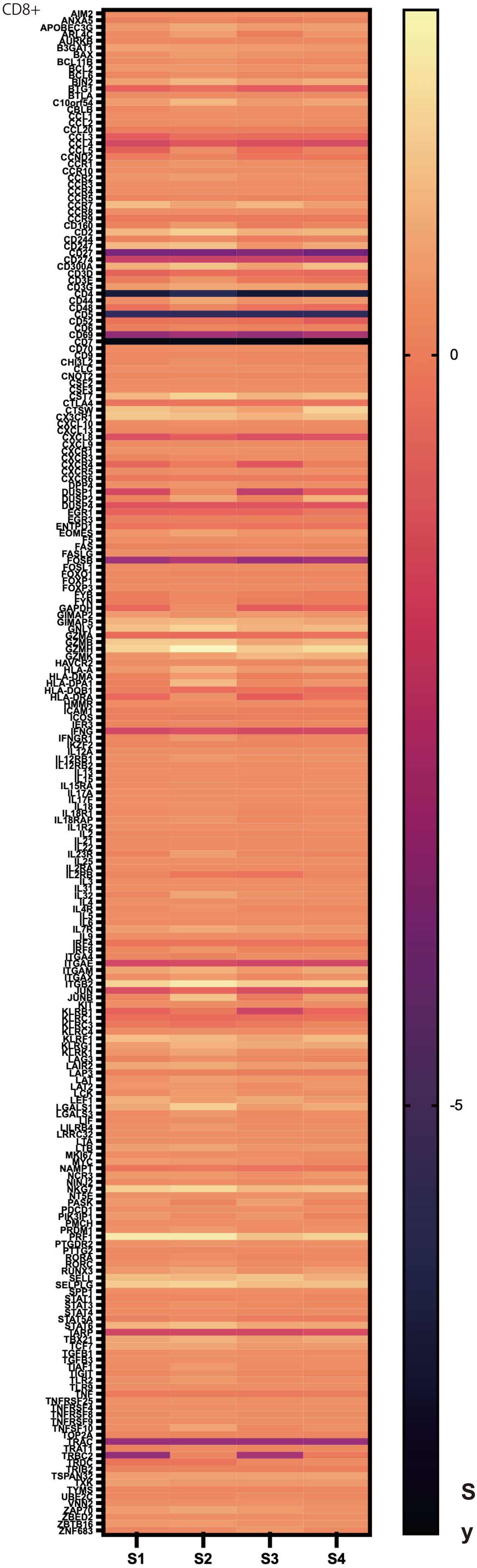

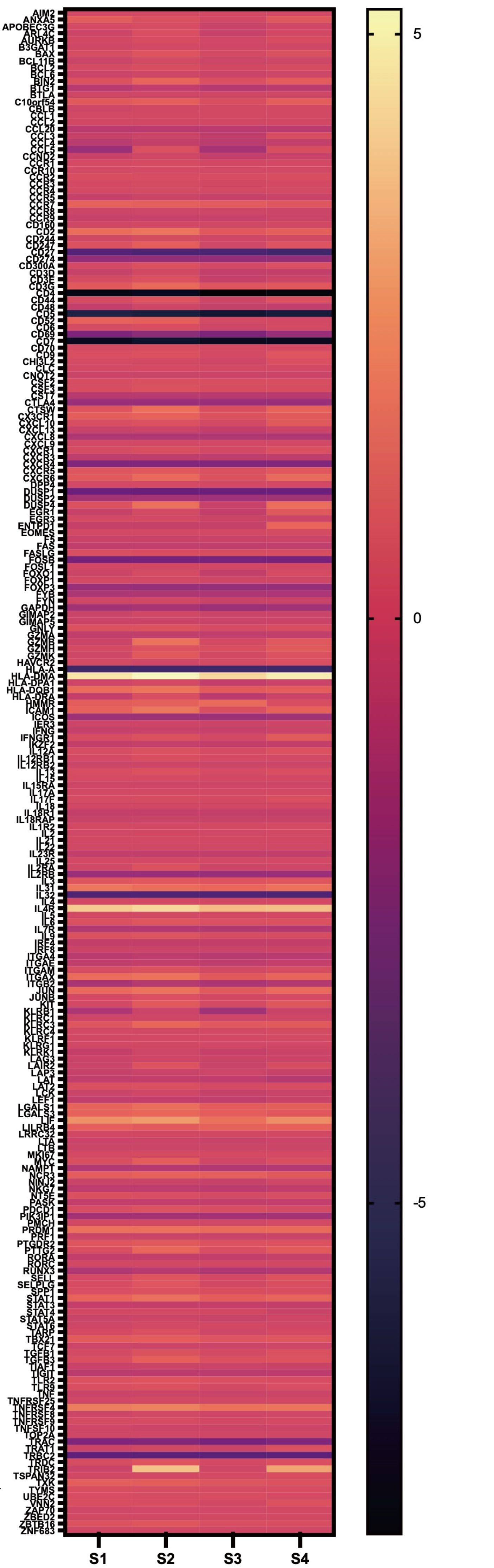

Supplementary Figure S3

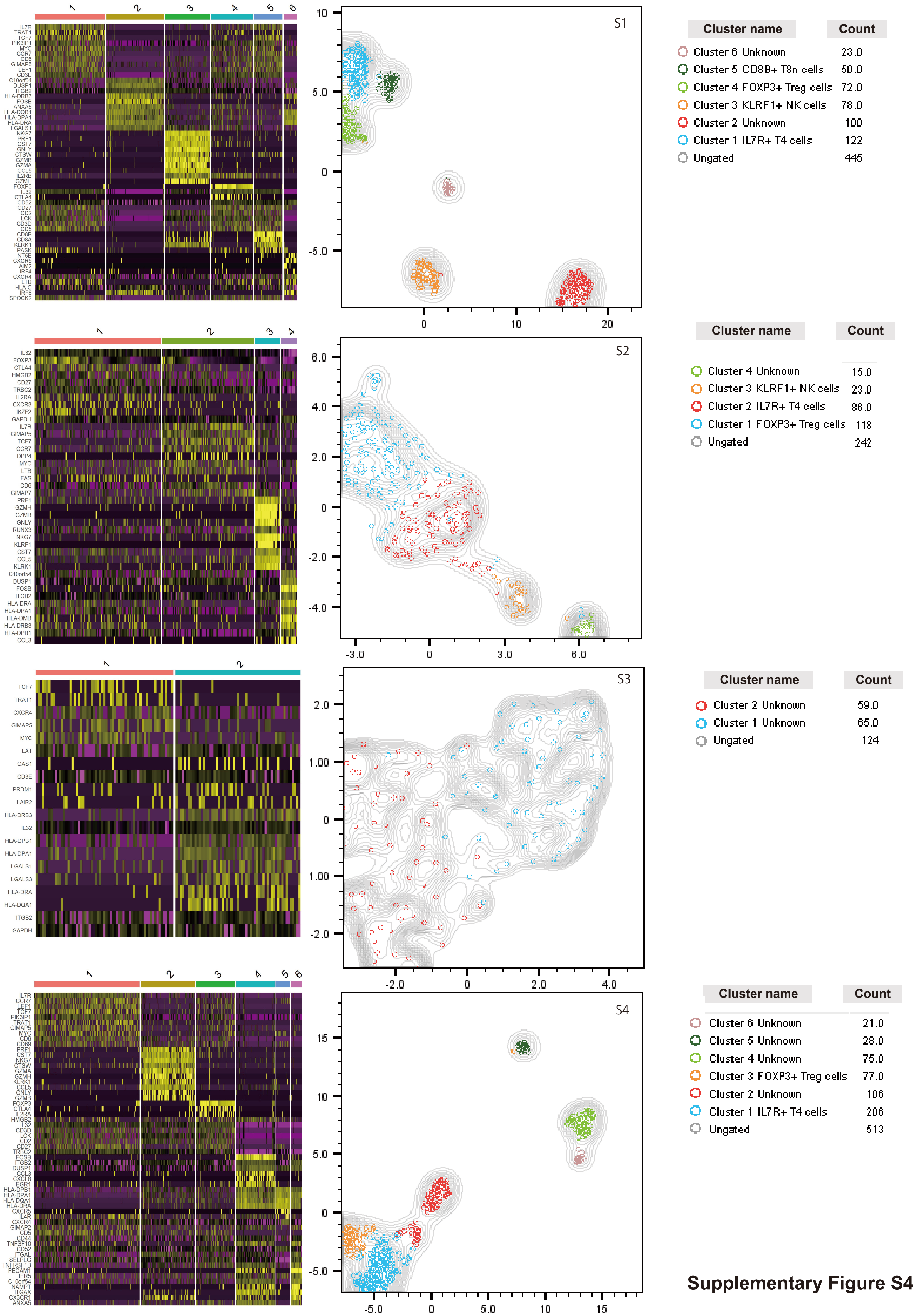

Supplementary Figure S4

A TRA CD8+ cells

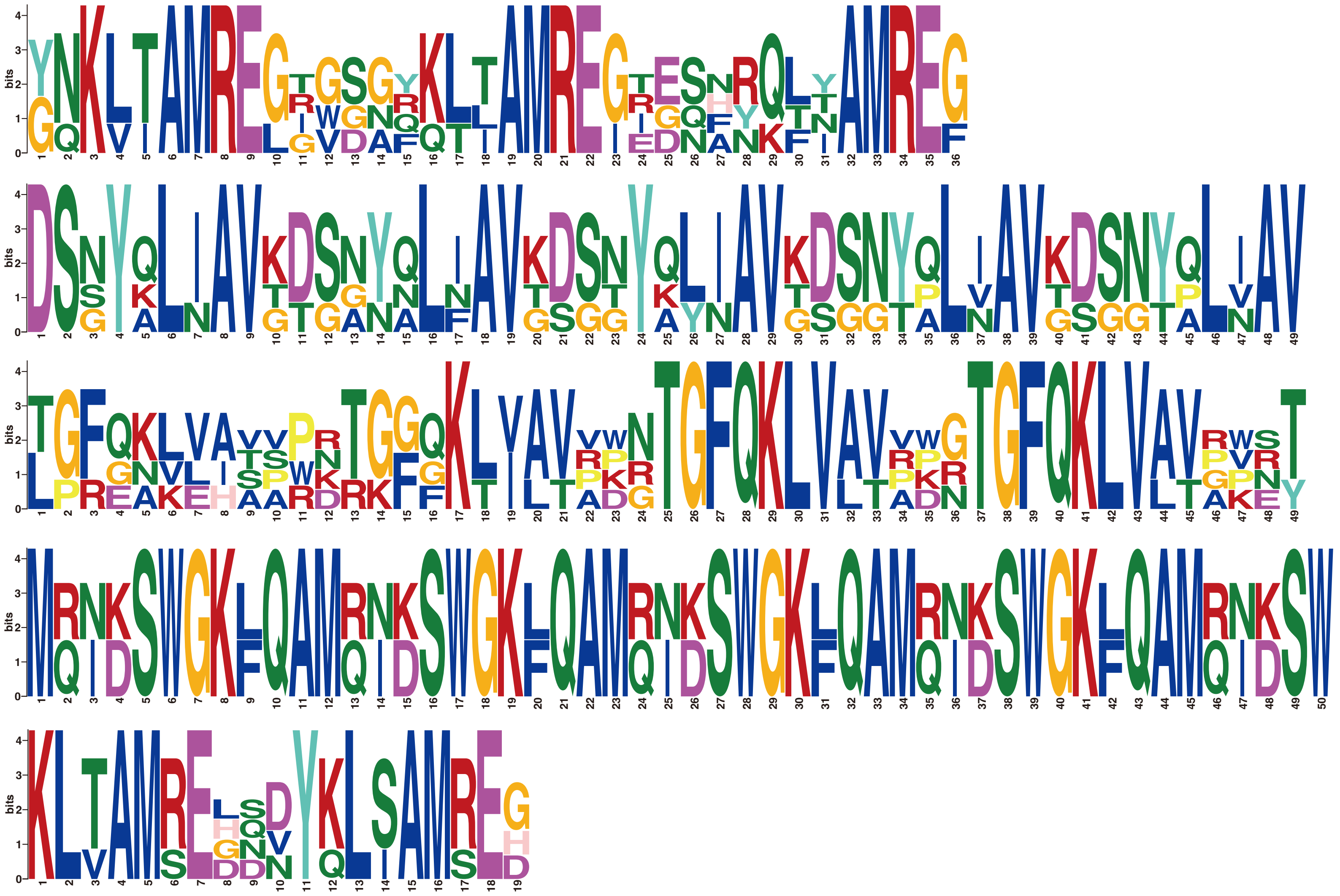

B TRB CD8+ cells

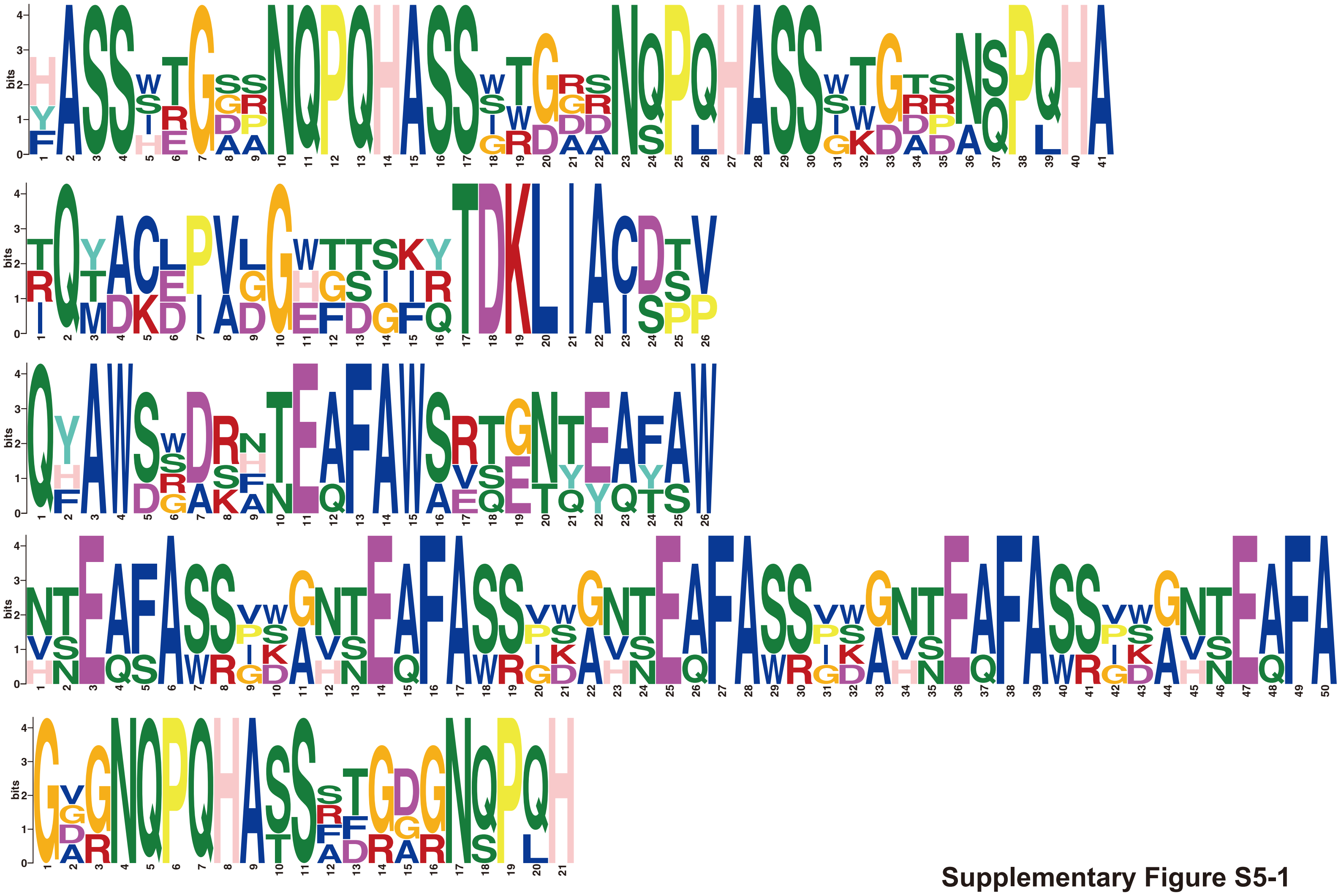

Supplementary Figure S5-1

C TRA FOXP3+ cells

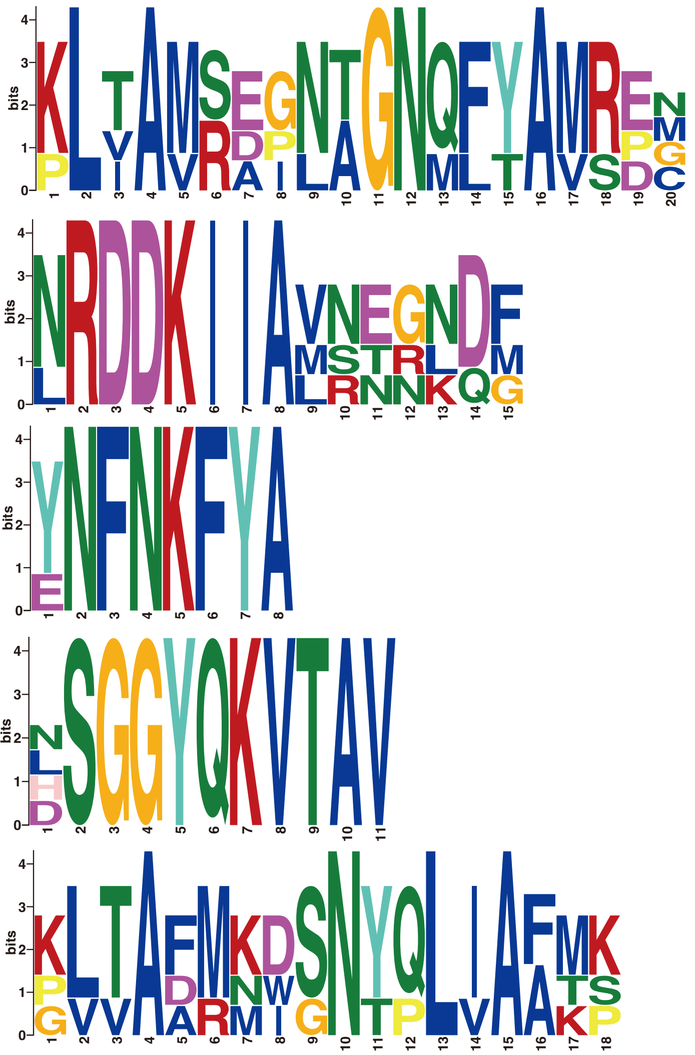

D TRB FOXP3+ cells

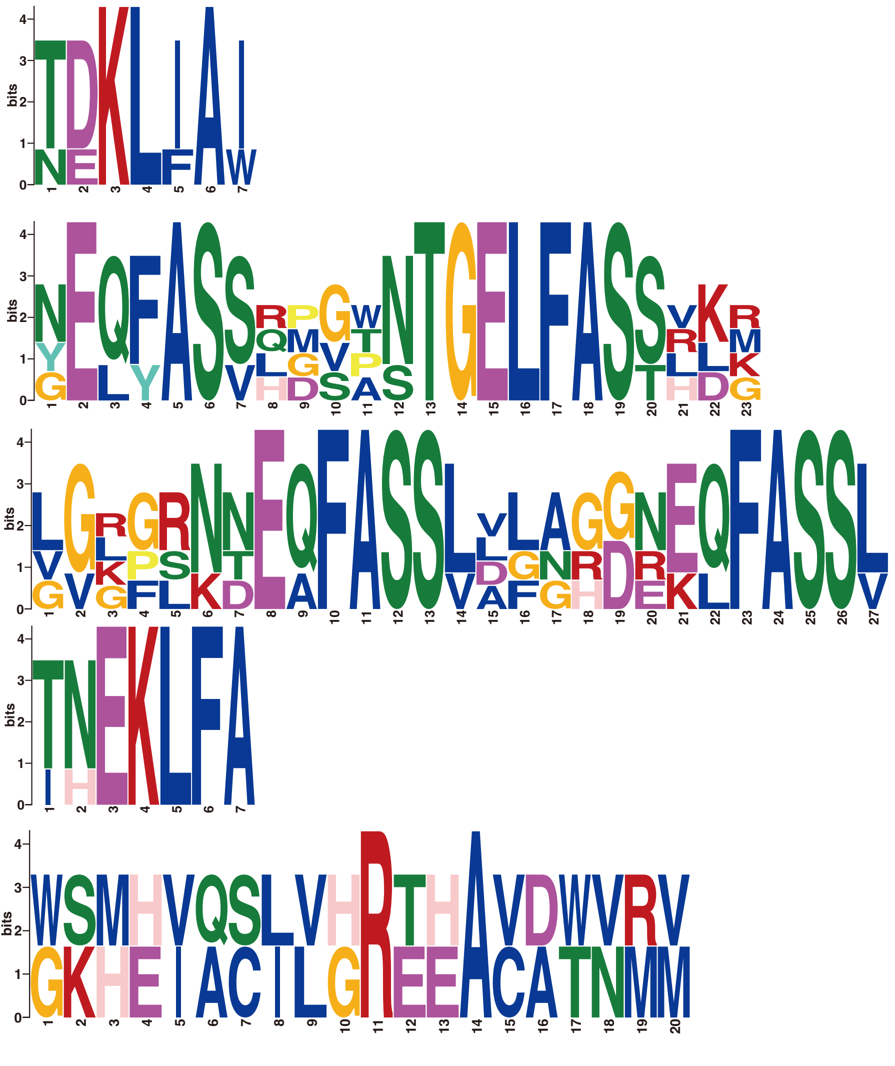

Supplementary Figure S5-2
